# The Use of an Intra-Articular Depth Guide in the Measurement of Partial Thickness Rotator Cuff Tears

**DOI:** 10.1155/2013/959305

**Published:** 2013-02-21

**Authors:** Michael J. Carroll, Kristie D. More, Stephen Sohmer, Atiba A. Nelson, Paul Sciore, Richard Boorman, Robert Hollinshead, Ian K. Y. Lo

**Affiliations:** ^1^Section of Orthopedic Surgery, Department of Surgery, University of Calgary, 3280 Hospital Drive, Calgary, AB, Canada T2N 4Z6; ^2^Sport Medicine Centre, University of Calgary, 2500 University Drive NW, Calgary, AB, Canada T2N 1N4; ^3^University of British Columbia, 301-909 Island Highway, Campbell River, BC, Canada V9W 2C2

## Abstract

*Purpose*. The purpose of this study was to compare the accuracy of the conventional method for determining the percentage of partial thickness rotator cuff tears to a method using an intra-articular depth guide. The clinical utility of the intra-articular depth guide was also examined. *Methods.* Partial rotator cuff tears were created in cadaveric shoulders. Exposed footprint, total tendon thickness, and percentage of tendon thickness torn were determined using both techniques. The results from the conventional and intra-articular depth guide methods were correlated with the true anatomic measurements. Thirty-two patients were evaluated in the clinical study. *Results*. Estimates of total tendon thickness (*r* = 0.41, *P* = 0.31) or percentage of thickness tears (*r* = 0.67, *P* = 0.07) using the conventional method did not correlate well with true tendon thickness. Using the intra-articular depth guide, estimates of exposed footprint (*r* = 0.92, *P* = 0.001), total tendon thickness (*r* = 0.96, *P* = 0.0001), and percentage of tendon thickness torn (*r* = 0.88, *P* = 0.004) correlated with true anatomic measurements. Seven of 32 patients had their treatment plan altered based on the measurements made by the intra-articular depth guide. *Conclusions*. The intra-articular depth guide appeared to better correlate with true anatomic measurements. It may be useful during the evaluation and development of treatment plans for partial thickness articular surface rotator cuff tears.

## 1. Introduction

Rotator cuff tears are a common cause of pain and disability in the adult shoulder [1], and surgery for the rotator cuff is one of the most common procedures performed in the shoulder. Rotator cuff tears may be divided into partial thickness tears and full thickness tears. Full-thickness tears involve complete disruption of the tendon thickness and are commonly treated with open or arthroscopic rotator cuff repair [1].

In clinical and cadaveric studies, partial thickness tears are approximately twice as common (6–39% incidence) as full-thickness tears [2–5]. These tears involve only partial disruption of the tendon thickness, and a portion of the tendon insertion remains intact on its footprint. Codman [3] first described partial thickness tears over seventy-five years ago. However, it was not until the development of arthroscopy that partial thickness tears became a commonly diagnosed and treated condition [2, 4, 5].

It is generally believed that the extent of partial thickness tearing correlates with the severity of symptoms [3, 5]. Articular surface tears are commonly agerelated and may be due to degeneration, while bursal surface tears are felt to be secondary to extrinsic impingement. Furthermore, several authors have recommended different treatment regimens for partial-thickness rotator cuff tears based on the tendon thickness involved. For example, Weber [6, 7] has recommended that partial-thickness rotator cuff tears involving >50% of the tendon thickness should be repaired. While this recommendation has become the standard of care [2, 4, 5], determining the extent of partial thickness rotator cuff tears remains difficult and imprecise. Weber in his study [7] noted that “objective means of assessing the depth of tear have proved elusive” and further Ruotolo and colleagues [8] in 2004 stated that “…, to date, no technique to accurately measure the thickness of a supraspinatus partial-thickness rotator cuff tear has been described.”

A conventional method may be employed to determine the percentage of partial thickness tear. It begins with an estimate of the amount of exposed bone in the rotator cuff footprint based on relative shaver diameter. This information is then related to historic anatomic data as well as clinical (e.g., gender, stature) and radiographic data to determine a percentage thickness tear [8, 9]. Considering the variability of footprint anatomy [8], this technique may lead to imprecise estimations of partial thickness rotator cuff tears and potentially incorrect treatment. Since partial thickness tears are so common, a simple and accurate method of measuring tendon thickness is required. The purpose of this study was to compare the accuracy of the conventional method of determining the percentage of partial thickness rotator cuff tears to a new method using an intra-articular depth guide in a cadaveric model. We also characterize and describe utility of the intra-articular depth guide in a clinical setting.

## 2. Methods

### 2.1. The Intra-Articular Depth Guide

The intra-articular depth guide (Arthrex, Inc., Naples, FL) is a simple tool used to quantify the percentage of tendon involved in partial thickness rotator cuff tears. This tool can be used to measure both bursal surface and articular surface tears. This study is limited to articular surface tears of the supraspinatus tendon.

**Figure 1:**
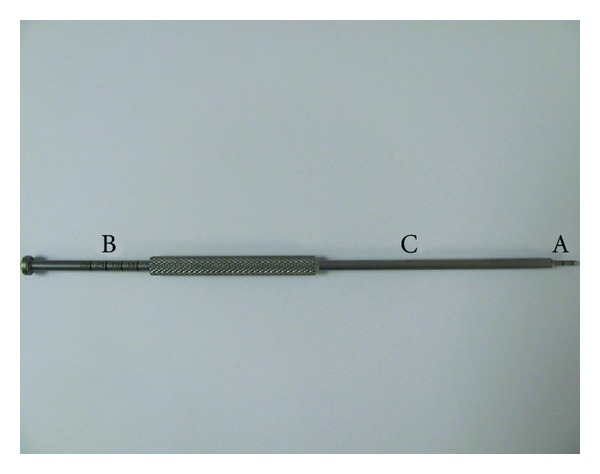
The assembled intra-articular depth guide. A: needle-tipped inner metallic probe used to penetrate the rotator cuff and measure the exposed bone bed. B: the distal end of the inner metallic probe is marked with numbers to measure total tendon thickness. C: outer metallic sleeve slides freely over the inner metallic probe.

The depth guide is designed and used similar to a standard depth gauge. It is a two-piece device with an inner “needle-tipped” metallic probe and an outer metallic sleeve, which slides freely over top (Figure 1). The inner probe measures the exposed footprint, while the outer sleeve determines the total tendon thickness.

**Figure 2:**
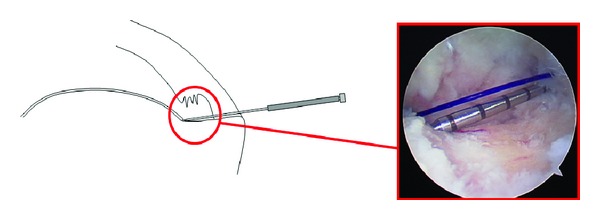
Schematic diagram and corresponding arthroscopic photo (of a right shoulder from a posterior glenohumeral portal) demonstrating the inner metallic sleeve penetrating the intact portion of the rotator cuff laterally at the area of greatest tendon involvement. The “needle tipped” portion of the intra-articular depth guide is inserted tangential to the bone bed and placed adjacent to the articular cartilage. (Note: all arthroscopic photos are oriented in the beach chair position).

To obtain an accurate measurement, the partial tear is first debrided on its articular surface. The tip of the inner metallic probe is then advanced through the remaining intact portion of the rotator cuff (Figure 2). Care is taken to ensure the depth guide is introduced tangential to the greater tuberosity with the tip of the inner metallic tube placed at the margin of the articular cartilage. The tip is marked by 3 mm lines and allows direct measurement of the exposed tendon footprint.

**Figure 3:**
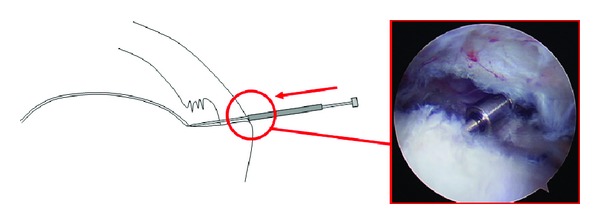
Schematic diagram and corresponding arthroscopic photo (of a right shoulder from a posterior subacromial portal) demonstrating advancement of the outer metallic sleeve to the bursal surface of the rotator cuff. The position of the outer sleeve on the inner metallic rod corresponds to the total tendon thickness.

**Figure 4:**
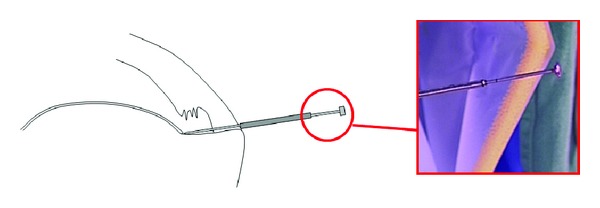
Schematic diagram and corresponding photo demonstrating that total tendon thickness can be read off the numbers on the distal portion of the inner metallic rod extracorporeally.

The outer metallic sleeve is then advanced through the deltoid until it is flush with the bursal surface of the rotator cuff tendon (Figure 3). The arthroscope may be placed in the subacromial space to ensure there is no interposed soft tissue between the tendon and guide. The total tendon thickness can then be read directly on the guide (Figure 4).

### 2.2. Cadaveric Study Design

The purpose of this study was to compare the accuracy of the conventional method of estimating the percentage of partial thickness rotator cuff tears to an intra-articular depth guide in a cadaveric model. The design was reviewed and approved by the institutions ethics committee. The conventional method and the technique of the intra-articular depth guide are described above. 

Twelve fresh frozen, unpaired cadaveric shoulders were identified for use in this study. In keeping with the scope of the study, specimens with full thickness rotator cuff tears were excluded. As such, four shoulders were removed from analysis. Cadavers with partial thickness articular surface tears of the rotator cuff were included. The mean age of the cadavers used was 71 years (range: 42–86); there were 6 female and 2 male patients.

Cadavers were mounted in the beach-chair position, and standard anterior and posterior glenohumeral portals were created with the pump maintaining pressure at 70 mmHg. In eight fresh frozen cadaveric shoulders, partial thickness articular surface tears of various amounts were created in the supraspinatus tendon with 5.5 mm shaver (Conmed Linvatec, Largo, FL). Existing partial tears underwent further debridement if required; the goal was to create tears involving approximately 33–67% of the total tendon thickness.

Each of the eight shoulders then underwent arthroscopic evaluation by two fellowship trained arthroscopic surgeons who were familiar with the depth guide. The extent of the partial thickness tear was estimated using the two techniques described. The shoulders were then dissected and a digital caliper (Mitutoyo, Japan) was used to determine the true amount of exposed footprint and total tendon thickness.

### 2.3. Clinical Study Design

The purpose of the clinical study was to characterize and describe the utility of the intra-articular depth guide in a clinical setting. From the period of January 2005–March 2006, all patients with partial thickness articular surface tears of the rotator cuff were evaluated.

All partial thickness articular surface tears were evaluated arthroscopically using both the conventional and intra-articular depth guide techniques.

### 2.4. Statistical Analysis

Absolute values for amount of footprint exposed and total tendon thickness were collected from (1) the conventional method, (2) intra-articular depth guide, and (3) true anatomic dissection in 8 shoulders.

The percentage of tendon thickness torn was determined for each group. It was calculated using the formula:

(1) Percentage  of  tendon  thickness  tear  =Exposed  footprintTotal  tendon  thickness×100%.

Mean amount of footprint exposed, total tendon thickness, and the percentage of thickness torn from the conventional method and intra-articular depth guide were compared to the true anatomic dissections using the Pearson correlation coefficient (*r*).

The mean difference in the percentage of tendon thickness torn using the two methods was also evaluated using a paired *t*-test. A *P* value < 0.05 was considered statistically significant.

## 3. Results 

### 3.1. Cadaveric Study

In the cadaveric study, the mean true amount of footprint exposed was 6.9 mm (range: 5–10 mm), the mean true total tendon thickness was 13.6 mm (range: 11 mm–22 mm), and the mean true percentage of tendon thickness torn was 54% (range: 27–72%). The results for the two methods of measurement as well as the true anatomic measures are summarized in Table 1.

Using the conventional method, the amount of exposed footprint correlated well with the true exposed footprint (*r* = 0.83, *P* = 0.01). However, total tendon thickness (*r* = 0.41, *P* = 0.31) and percentage of thickness torn (*r* = 0.67, *P* = 0.07) did not significantly correlate with the true anatomic measurements.

The intra-articular depth guide and true anatomical measurements correlated well in all categories. Statistical significance was achieved with the amount of exposed footprint (*r* = 0.92, *P* = 0.001), total tendon thickness (*r* = 0.96, *P* = 0.0001), and percentage of tendon thickness torn (*r* = 0.88, *P* = 0.004).

The difference in mean percentage of tendon thickness torn between the conventional method and true anatomic measurement was 15% ± 12% (range: 2%–27%). The difference between the intra-articular depth guide and true anatomic measurement was 7% ± 4% (range: 3%–13%). There was no statistically significant difference between the conventional method and intra-articular depth guide (*P* = 0.09).

### 3.2. Clinical Study

From January 2005 to March 2006, 32 patients with partial thickness tears of the supraspinatus tendon were evaluated both using the conventional method and the intra-articular depth guide. Inclusion criteria were male and female patients with partial thickness articular surface tears involving the supraspinatus tendon. The mean age of the patients was 50 ± 9.4 years with 9 female and 23 male patients involving 4 nondominant and 28 dominant shoulders. In 27 patients the primary diagnosis was a partial thickness tear of the rotator cuff, 3 patients had superior labral tears, 1 had adhesive capsulitis, and in 1 patient the primary diagnosis was osteoarthritis.

Using the conventional method, the amount of footprint exposed was estimated relative to shaver diameter. The mean exposed footprint was 5.1 mm (range: 0–18 mm). The surgeon estimated the total tendon thickness by taking a number of factors into consideration. Using known historical data in conjunction with the results of preoperative imaging (e.g., ultrasound, MRI, MRI arthrogram), clinical features (e.g., male/female, size of patient), and intraoperative findings (e.g., comparison to a known size instrument), an estimate was made. The mean total tendon thickness was 13.7 mm (range: 10–20 mm), and the mean percentage of thickness torn was 36.8% (0–90%).

Using the intra-articular depth guide, the mean footprint exposed was 6.2 mm (0–17 mm), the mean total tendon thickness was 16.4 mm (range: 13–22 mm), and the mean percentage of thickness torn was 38.1% (range: 0%–94%). The mean difference between the two methods for percentage of thickness torn was 6.8% (range: 0–26%).

The difference was significant enough clinically to alter the treatment decision for 7 patients when using 50% of the tendon thickness torn as a guide. In 4 cases, when using the intra-articular depth guide the tendon was determined to be <50% torn and was debrided instead of repaired; in 3 cases the tendon was found to be >50% torn and was repaired instead of undergoing debridement. Repairs were performed using a transtendon technique as previously described [10]. In brief, anchors were inserted transtendon along the medial margin of the footprint, and sutures were passed through the intact rotator cuff tendon using a mattress configuration. Sutures were then tied in the subacromial space to compress the tendon against the medial aspect of the footprint.

## 4. Discussion

This study describes a new method to quantify percentage of partial thickness rotator cuff tendon tears using an intra-articular depth guide. The guide enables the surgeon to accurately determine the amount of exposed footprint and the total tendon thickness. The technique is relatively simple and to our knowledge is the only method to directly measure the total tendon thickness and determine the percentage of thickness tears arthroscopically.

Measuring the amount of exposed footprint may be determined relatively accurately using both the conventional method or intra-articular guide. As compared to the conventional method of quantifying percentage of partial thickness tears (i.e., estimating amount of exposed footprint with reference to a shaver of known size), the intra-articular depth guide appeared to better correlate to true anatomic measurements. This finding is largely related to the accuracy in which the depth guide can determine total tendon thickness.

The significance of this finding relates to the fact that treatment decisions tend to be based on the percentage of tendon that is torn. Though controversial, there is a general consensus that partial thickness tears involving >50% of the tendon thickness should be repaired [2, 4–8]. Despite this, the percentage of partial thickness tears is inferred since no alternative method of quantifying them has been described. The conventional method relies on estimating the amount of footprint exposed and incorporating historical anatomic data. We have shown that it may be prone to error and may not correlate well with true anatomic measurements.

The current standard of treatment (using 6 mm of exposed bone bed) arbitrarily compares the amount of exposed bone in the rotator cuff footprint, to historical anatomical data to determine the percentage of tendon involvement. Recently, Ruotolo et al. [8] demonstrated that the mean medial to lateral thickness of the supraspinatus tendon insertion was approximately 12 mm and recommended that all tears with more than 7 mm of exposed bone lateral to the articular surface (i.e., 6 mm of exposed footprint) representing “>50% of the tendon substance” should be repaired.

Although this method may be applied in general, the medial to lateral thickness of the tendon in Nottage's study was remarkably variable and ranged from 8 to 15 mm [8]. This means that the 6 mm of exposed footprint recommendation may represent anywhere from 37 to 69% of the tendon thickness. Curtis and Tierney [9] provide further support and demonstrated in another cadaveric study that the supraspinatus tendon footprint was also quite variable in size. The average width was 16 mm, yet the tendon thickness ranged from 12 mm to 22 mm. Therefore, 6 mm of exposed bone bed could represent 27–50% of the total tendon thickness.

The intra-articular depth guide provides an accurate method of determining the amount of footprint exposed, total tendon thickness, and percentage of partial thickness tears. This information was utilized in our clinical series when 7 of 32 patients (22%) had their treatment plan modified based on measurements taken using the intra-articular depth guide when compared to the conventional method. Larger clinical studies in which treatment plans are designed using both techniques will provide additional information on the utility of the intra-articular depth gauge.

We acknowledge certain limitations to our study. While we acknowledge the sample size was small in both the cadaveric and clinical studies in the purpose of the study was to determine the accuracy of the intra-articular depth guide and we believe the results are significant and relevant. Furthermore, the inter- and intraobserver reliability of the intra-articular depth guide was not determined. We also described the clinical application of the intra-articular depth gauge in a small cohort of patients. In the description of patients, the true anatomic measurements were unknown. While confirming with open repair or dissection to determine the “true” percentage of the partial thickness tearing would have provided additional information, this would subject patient to unnecessary morbidity.

## 5. Conclusion

As compared to the conventional method of quantifying the percentage of partial thickness tears, the intra-articular depth guide appeared to better correlate to true anatomic measurements. This method may be useful during the evaluation and development of treatment plans for partial thickness articular surface rotator cuff tears. Larger clinical studies looking at patient outcomes are required to better characterize the utility of the intra-articular depth gauge in formulating treatment plans.

## Figures and Tables

**Table 1:** Measurements of the exposed footprint, total tendon thickness, and percentage of tendon thickness torn in cadaveric shoulders with simulated partial thickness articular surface rotator cuff tears using the methods of comparison to a known size, the intra-articular depth guide, and true anatomic measures.

	Conventional technique		Depth guide		True anatomic measure	
	Mean	Range	Mean	Range	Mean	Range
Exposed footprint	5.5 mm	4 mm–8 mm	6.8 mm	5 mm–10 mm	6.9 mm	5 mm–10 mm
Total tendon thickness	14.5 mm	10 mm–18 mm	13.5 mm	11 mm–20 mm	13.6 mm	11 mm–22 mm
Percentage of thickness torn	39%	22%–50%	52%	30%–76%	54%	27%–72%
